# True mineral digestibility in C57Bl/6J mice

**DOI:** 10.1371/journal.pone.0290145

**Published:** 2023-08-16

**Authors:** Linda F. Böswald, Jasmin Wenderlein, Wolfgang Siegert, Reinhard K. Straubinger, Ellen Kienzle

**Affiliations:** 1 Chair for Animal Nutrition and Dietetics, Department of Veterinary Sciences, Faculty of Veterinary Medicine, LMU München, München, Germany; 2 Chair of Bacteriology and Mycology, Institute of Infectious Diseases and Zoonoses, Department of Veterinary Sciences, Faculty of Veterinary Medicine, LMU München, München, Germany; 3 Institute of Animal Science, University of Hohenheim, Stuttgart, Germany; RWTH Aachen University Medical Faculty: Rheinisch-Westfalische Technische Hochschule Aachen Medizinische Fakultat, GERMANY

## Abstract

Data on mineral digestibility is key to understand mineral homeostasis and refine the recommendations for the dietary intake of these nutrients. In farm animals and pets, there is plenty of data on mineral digestibility and influencing factors. In laboratory mice, however, there is a lack of information on mineral digestibility under maintenance conditions, although this should be the basis for studies on mineral homeostasis under experimental conditions. The aim of the present study was to analyse data on intake, faecal excretion, and apparent digestibility of calcium, phosphorus, sodium, potassium, and magnesium in C57BL/6J mice fed different maintenance diets with varying voluntary dry matter intake. Lucas-tests were used to quantify true digestibility and describe correlations between dietary intake and excretion/absorption of the nutrients. Calcium, phosphorus, and magnesium showed a linear correlation between intake and faecal excretion (R^2^: 0.77, 0.93 and 0.91, respectively). Intake and apparently digested amounts of sodium and potassium were correlated linearly (R^2^: 0.86 and 0.98, respectively). These data show that intake is the major determinant of absorption in the minerals listed above. Faecal calcium and phosphorus excretion were correlated as well (R^2^ = 0.75).

## Introduction

Studies on quantitative aspects of mineral homeostasis have been conducted in several species. In dogs and cats, it was shown that calcium digestibility is mainly determined by calcium intake [1, 2] and not, as previously thought, regulated closely depending on the calcium status of the organism. Kienzle & Burger [3] collected literature data on intake and faecal excretion of major minerals in horses to estimate the true digestibility for these nutrients. Such data is invaluable for factorial calculation of nutrient requirements [4–6] and may show whether other dietary factors influence mineral digestibility [1]. For the factorial calculation of nutrient requirements, it is necessary to establish a dataset of intake and excretion of the nutient in question. When a broad range of nutrient intake (from low and marginal intake to excessively high) is covered, the inevitable endogenous losses can be estimated reliably by extrapolating the excretion at zero intake. The endogenous losses and the mean availability of the nutrient are used to caluclate the “gross” requirement, i.e. the amount to be fed.

While extensive literature exists on the estimation of nutrient requirements in pet and farm animal species, in laboratory mice, the recommendations for nutrient supply are based on a limited number of studies, emprical data and partly extrapolation from rats [7]. The factorial calculation of mineral requirements would yield more exact and species-specific values. Considering the 3R principles [8], an adequate and species-specific nutrition must be key to refining laboratory mouse husbandry and welfare. It is also essential to ensure the quality of animal experiments. However, there is a lack of data on mineral digestibility under non-experimental conditions, so that no dataset for a factorial calculation of the mineral requirement is available to the authors´ knowledge. The first step in this direction is the investigation of apparent digestibility of minerals in mice fed diets with sufficient mineral supply. Such data can be quantitatively evaluated with the Lucas-test, or where applicable its modified form [1, 2, 9, 10], to identify patterns or influencing factors on apparent digestibility. In further steps, for each nutrient digestibility trials with graded concentrations below and above the normal nutrient supply need to be conducted.

With the data reported in the present study, we aim to make the first step to broaden the knowledge on mineral digestibility in laboratory mice. In order to reduce the number of animals used for experimental purposes, we analyzed samples from a previous digestibility trial [11] that had the primary goal to compare differently processed diets. The difference in starch gelatinization between pelleted and extruded diets led to the expected differences in energy digestibility and microbiome composition. In this trial, intake and digestibility of dry matter varied considerably (apparent dry matter digestibility 71–81%). In other species, an effect of dry matter digestibility on intestinal mineral uptake is known [12–14]. The dataset from the previous mouse experiment was therefore considered to be an ideal setting to search for potential effects of dry matter digestibility, fibre, and starch on mineral digestibility under standard conditions. The mouse strain used in the experiment, C57Bl/6J, is a common inbred strain that is widely used in many research areas [15]. Thus, any additional knowledge on physiological processes of these mice is of high interest.

The aim of the study was to evaluate the quantitative digestibility patterns of minerals in C57Bl/6J mice and to identify possible effects of the abovementioned dietary factors from data of a study on diet processing [11, 16].

## Materials and methods

The feeding trials have been designed with the aim to compare pelleted and extruded maintenance diets in regard of energy and crude nutrient digestibility [11] and gastrointestinal microbiome [16]. Ethical approval by the appropriate authorities was given (reference no. 169-03-05-2019). In the present retrospective evaluation, the mineral homeostasis was in the focus.

### Animals

In the feeding trials, 54 female C57Bl/6J mice in maintenance metabolism were used (eight weeks old, bred by Envigo RMS B.V., Netherlands). The mice were housed in groups of 2–3 mice in isocages (Techniplast, Buguggiate, Italy) under specified-pathogen-free (SPF) conditions on silicate bedding (Tigerino Crystals, Matina GmbH, Munich, Germany). Two trials (Trials #1 and #2) were conducted consecutively and followed the procedure as described in previous publications [11, 16] (study protocol approved by the Committee of the Faculty of Veterinary Medicine, LMU München, protocol code 169-03-05-2019). They did not involve any invasive procedures for the animals. The mice were sacrified by cervical dislocation after the experiment.

### Diets

Commercial maintenance diets for laboratory mice by the same manufacturer were used. According to the manufacturer´s information, the diets differed only in processing, not raw materials, and were thus chosen for the original study. For the present evaluation, the diets were named according to processing (pelleted / extruded) and mean apparent dry matter digestibility [11]: PEL_71_, EXT_77_, PEL_81_, EXT_78_. Dietary mineral content is listed in Table 1. There were slight and non-systematic differences in the mineral contents. Diet PEL_81_ contained more starch (43%) and less total dietary fibre (16%) than the other diets (27–28% and 23–28%, respectively; as-fed basis). Further details on the diets and the corresponding energy and crude nutrient digestibility were published previously [11]. The diets were fed *ad libitum* after autoclaving them into the SPF facility. Water was freely available throughout the experiment. The mice were weighed weekly.

**Table 1:** Dry matter and mineral content of the diets used in Trial #1 and #2.

Nutrient	PEL_71_	EXT_77_	PEL_81_	EXT_78_
	*% as fed*			
Dry matter	89.9	89.9	89.5	88.5
	*% dry matter*			
Crude fibre	6.7	9.1	5.9	6.5
Starch	30.4	30.1	49.0	31.6
Calcium	0.79	0.76	0.71	0.83
Phosphorus	0.69	0.70	0.56	0.72
Sodium	0.25	0.26	0.33	0.34
Potassium	1.34	1.21	1.02	1.38
Magnesium	0.26	0.28	0.22	0.36
Inositol phosphates	*μmol/g dry matter*			
Ins(1,2,3,4,6)P	< 0.3	< 0.3	0.4	< 0.3
Ins(1,2,3,4,5)P	0.5	0.5	0.3	0.7
Ins(1,2,4,5,6)P	0.9	1.1	0.6	1.2
InsP_6_ (phytate)	15.9	15.5	11.6	19.5

### Experimental procedures

After an adaptation period to the diets of three weeks, daily feed intake was recorded by weighing the offered feed and the leftover amount after 24 hours. The total faecal mass was collected from the cages every day for 14–17 days. The faecal samples were stored at -20°C, then lyophilized, ground, and analysed for mineral content with standard methods (calcium, magnesium, potassium: flame emission photometry; phosphorus: photometry with ammonium molybdate and ammonium vanadate in HNO_3_ [17]; sodium: atomic absorption spectroscopy). Diet samples underwent the same mineral analyses. In addition, inositol phosphates were measured as described previously [18]. For each cage as a unit, the percentual apparent digestibility of the minerals was calculated with the following equation:

Apparentdigestibility(mineral)=(mineralintake–faecalmineralexcretion)/mineralintake*100

Mineral intake and faecal mineral excretion were calculated for each cage unit, divided by the number of animals per cage for values per animal and expressed in mg/kg body weight (BW). The mean BW per mouse was used.

### Statistics

The mean BW data passed the Shapiro Wilk normality test (*p* > 0.05) and was compared between the diet groups via one-way analysis of variance (significance level *α* = 0.05).

For the nutrients with an expectedly high apparent digestibility (sodium and potassium), the linear regression of intake and apparently digested amount (mg/kg BW) was calculated (Lucas-test [9]). In this model, the slope of the regression line * 100 equals the mean true digestibility of the nutrient (%). For the other minerals with lower apparent digestibility (calcium, phosphorus, magnesium), a modified Lucas-test was performed [2, 9] with correlation of intake and faecal excretion of the respective nutrient. In this case, (100 –slope) * 100 gives the true digestibility (%).

The fit of the regression equation was described with the coefficient of determination (R^2^) and the standard deviation of the residuals (Sy.x). A linear correlation was defined as “strong” with a coefficient of determination R^2^ ≥ 0.80. Diet groups were compared with a Kruskal-Wallis test, followed by a Dunn´s multiple comparison test because data was not normally distributed. A difference with *p* ≤ 0.05 was defined as significant. Statistics and graphic illustration were conducted with Graphpad Prism® 5.04 (Graphpad Software, San Diego, CA, USA).

## Results

There was a significant difference between the mean BWs of the diet groups (PEL_71_: 20.1 ± 1.6 g; EXT_77_: 20.6 ± 1.5 g; PEL_81_: 18.8 ± 0.9 g; EXT_78_: 18.8 ± 0.9 g; *p* < 0.05) that can be explained by energy intake and digestibility [11]. This does not impact the further results, because data was referenced to BW in all analyses.

Table 2 provides an overview of intake, apparently digested amount, apparent digestibility, and faecal excretion of each nutrient investigated. The results are detailed in the following sections for each nutrient.

**Table 2:** Overview of intake, apparently digested, and faecally excreted amount of the minerals as well as the apparent digestibility.

	*Unit*	PEL_71_	EXT_77_	PEL_81_	EXT_78_
**Calcium**					
Intake	*mg/kg BW*	1508^a^ ± 231	1444^a^ ± 186	1215^b^ ± 100	1699^a^ ± 109
Apparently digested	*mg/kg BW*	69^b,c^ ± 14	147^a,b^ ± 40	61^c^ ± 49	317^a^ ± 68
Faecally excreted	*mg/kg BW*	1439^a^ ± 226	1297^a,b^ ± 180	1154^b^ ± 113	1383^a^ ± 104
Apparent digestibility	*%*	5^b,c^ ± 1	10^a,b^ ± 3	5^c^ ± 4	19^a^ ± 4
**Phosphorus**					
Intake	*mg/kg BW*	1317^a^ ± 202	1337^a^ ± 173	945^b^ ± 78	1493^a^ ± 96
Apparently digested	*mg/kg BW*	397^a^ ± 86	495^a^ ± 63	282^b^ ± 40	462^a^ ± 56
Faecally excreted	*mg/kg BW*	919^a,b^ ± 120	843^b^ ± 123	663^c^ ± 58	1031^a^ ± 67
Apparent digestibility	*%*	30^b^ ± 2	37^a^ ± 3	30^b^ ± 3	31^b^ ± 3
**Sodium**					
Intake	*mg/kg BW*	434^c^ ± 37	456^c^ ± 55	559^b^ ± 46	689^a^ ± 44
Apparently digested	*mg/kg BW*	275^d^ ± 18	323^c^ ± 34	482^b^ ± 37	530^a^ ± 38
Faecally excreted	*mg/kg BW*	160^a^ ± 32	133^a^ ± 31	77^b^ ± 16	159^a^ ± 10
Apparent digestibility	*%*	64^c^ ± 5	71^c,b^ ± 4	86^a^ ± 2	77^b^ ± 1
**Potassium**					
Intake	*mg/kg BW*	2548^a,b^ ± 391	2314^b^ ± 298	1736^c^ ± 144	2848^a^ ± 183
Apparently digested	*mg/kg BW*	2003^a,b^ ± 360	1837^b^ ± 232	1462^c^ ± 107	2284^a^ ± 150
Faecally excreted	*mg/kg BW*	545^a^ ± 42	477^a^ ± 80	274^b^ ± 40	564^a^ ± 44
Apparent digestibility	*%*	78^b^ ± 2	79^b^ ± 2	84^a^ ± 1	80^b^ ± 1
**Magnesium**					
Intake	*mg/kg BW*	495^b^ ± 76	526^b^ ± 68	378^c^ ± 31	734^a^ ± 47
Apparently digested	*mg/kg BW*	166^a,b^ ± 78	170^a^ ± 27	117^b^ ± 12	197^a^ ± 35
Faecally excreted	*mg/kg BW*	329^b^ ± 23	356^b^ ± 45	262^c^ ± 22	536^a^ ± 45
Apparent digestibility	*%*	32^a,b^ ± 10	32^a^ ± 2	31^a,b^ ± 2	27^b^ ± 4

BW = body weight

Means in one row sharing the same superscript letter do not differ significantly (*p* < 0.05).

### Calcium

Calcium intake was significantly lower in PEL_81_ compared to all other diets (*p* < 0.001). The apparent digestibility of calcium was lower in the pelleted diets than the extruded diets.

**Fig 1:**
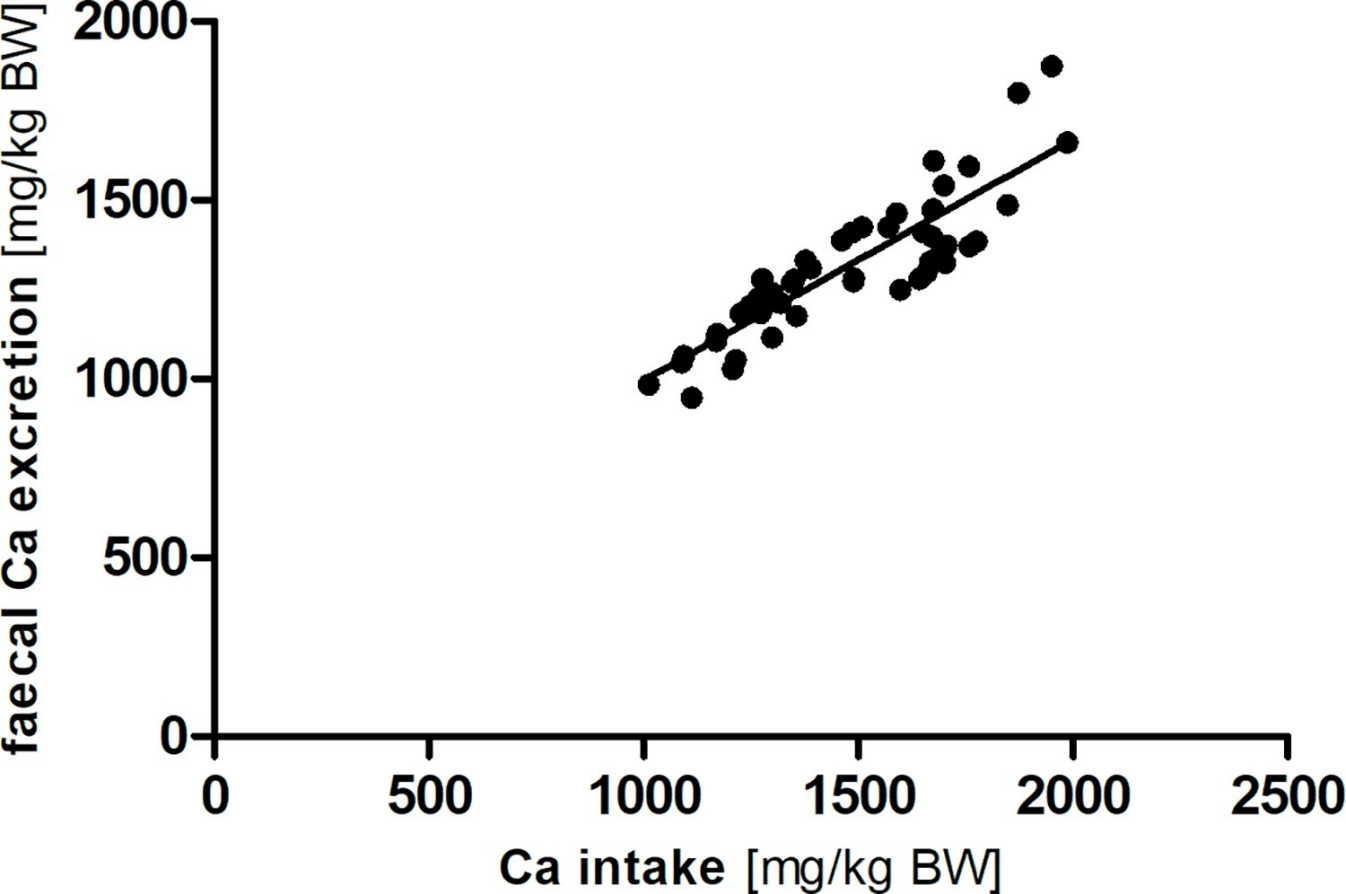
Linear correlation between calcium (Ca) intake and faecal excretion (y = 0.67 x + 321.91; R^2^ = 0.77; Sy.x = 91).

Fig 1 shows the linear correlation between calcium intake and faecal calcium excretion over all diet groups (R^2^ = 0.77, Sy.x = 91) in the modified Lucas-test.

### Phosphorus

The phosphorus intake was significantly lower in PEL_81_ than in the other diet groups (*p* < 0.001). Apparent phosphorus digestibility was significantly higher in EXT_77_ than in the other groups (*p* < 0.001) while the lowest absolute amount of apparently digested phosphorus (mg/kg BW) was found in group PEL_81_ (*p* < 0.05).

**Fig 2:**
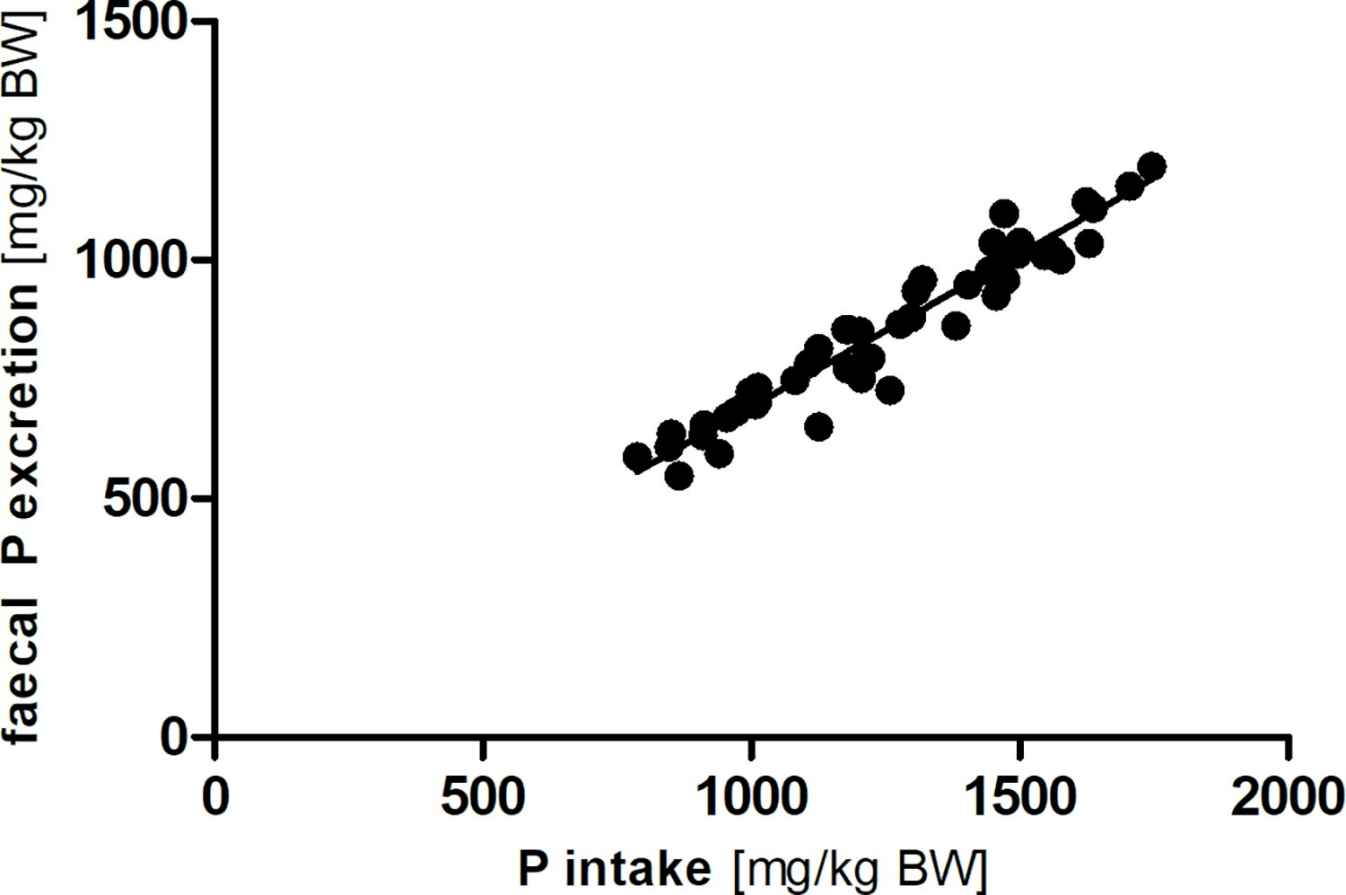
Linear correlation between phosphorus (P) intake and faecal excretion (y = 0.64 x + 53.88; R^2^ = 0.93; Sy.x = 47).

**Fig 3:**
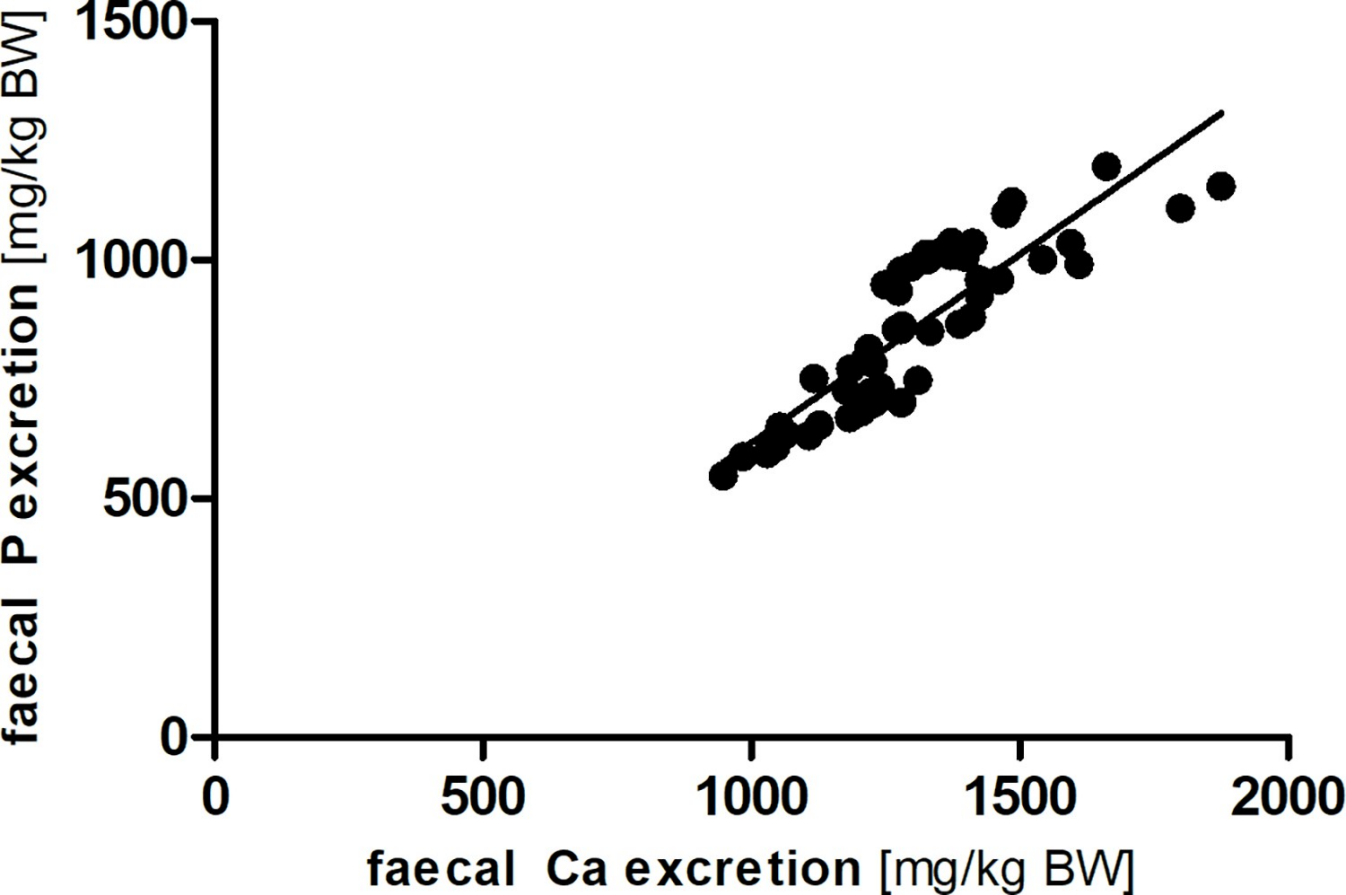
Linear correlation between faecal calcium (Ca) and phosphorus (P) excretion (y = 0.79 x– 171.07; R^2^ = 0.75; Sy.x = 87).

There was a strong linear relationship between phosphorus intake and faecal phosphorus excretion in the modified Lucas-test (R^2^ = 0.93; Sy.x = 47; Fig 2). In addition, faecal phosphorus and faecal calcium excretion were also correlated in a linear way (R^2^ = 0.75; Sy.x = 87; Fig 3).

### Sodium

**Fig 4:**
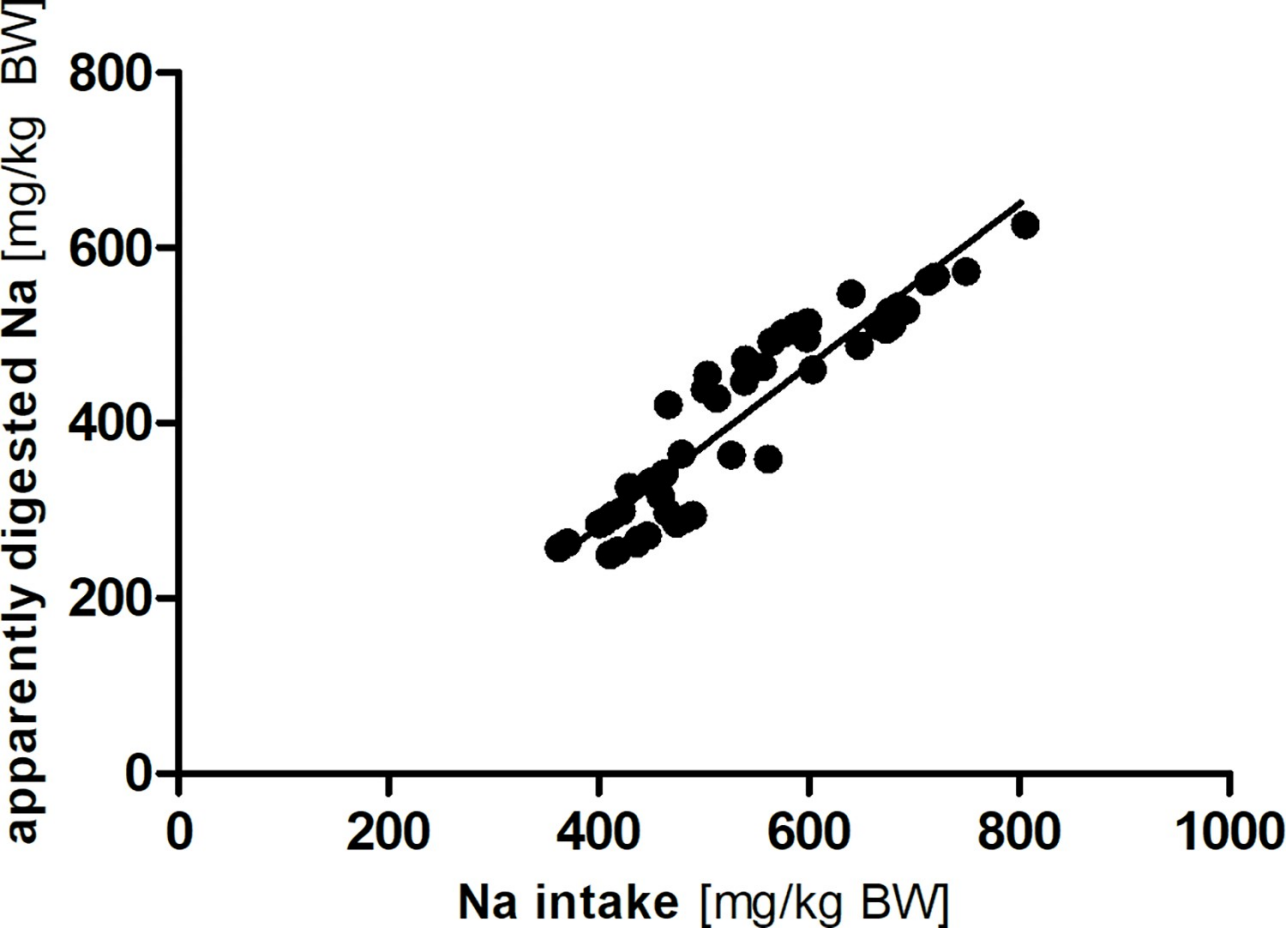
Linear correlation between sodium (Na) intake and apparently digested amount (y = 0.92 x– 85.70; R^2^ = 0.86; Sy.x = 41).

Sodium intake was lower in PEL_71_ and EXT_77_ than in PEL_81_ and EXT_78_ (Table 2; *p* < 0.05). There were differences among apparent digestibility, faecal excretion, and the apparently digested amount of sodium, however not systematic. There was a strong linear correlation between sodium intake and the amount of apparently digested sodium in the Lucas-test (mg/kg BW; R^2^ = 0.86; Sy.x = 41; Fig 4).

### Potassium

**Fig 5:**
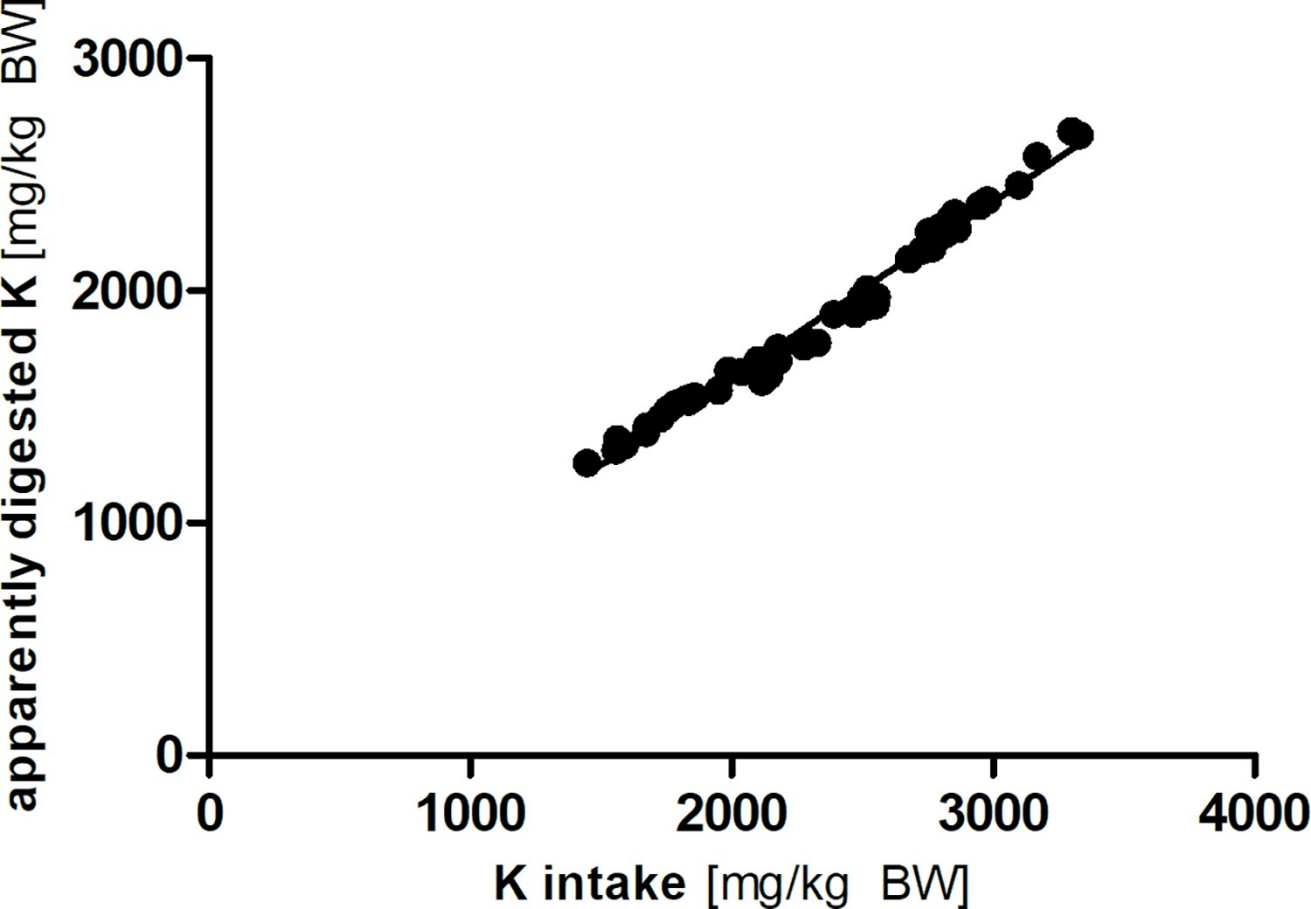
Linear correlation between potassium (K) intake and apparently digested amount (y = 0.76 x + 118.48; R^2^ = 0.98; Sy.x = 49).

Potassium intake differed between the groups, but not in a systematic way (see Table 2). Apparent potassium digestibility was significantly lower in diet PEL_81_ than the other diets (*p* < 0.05). Intake and apparently digested amount of potassium showed an extremely strong linear correlation in the Lucas-test (R^2^ = 0.98; Sy.x = 49; Fig 5).

### Magnesium

The mice fed PEL_81_ had a significantly lower amount of apparently digested Mg than the mice fed extruded diets (*p* < 0.001). The lowest apparent digestibility of Mg was found in diet EXT_78_ with 27 ± 4%.

**Fig 6:**
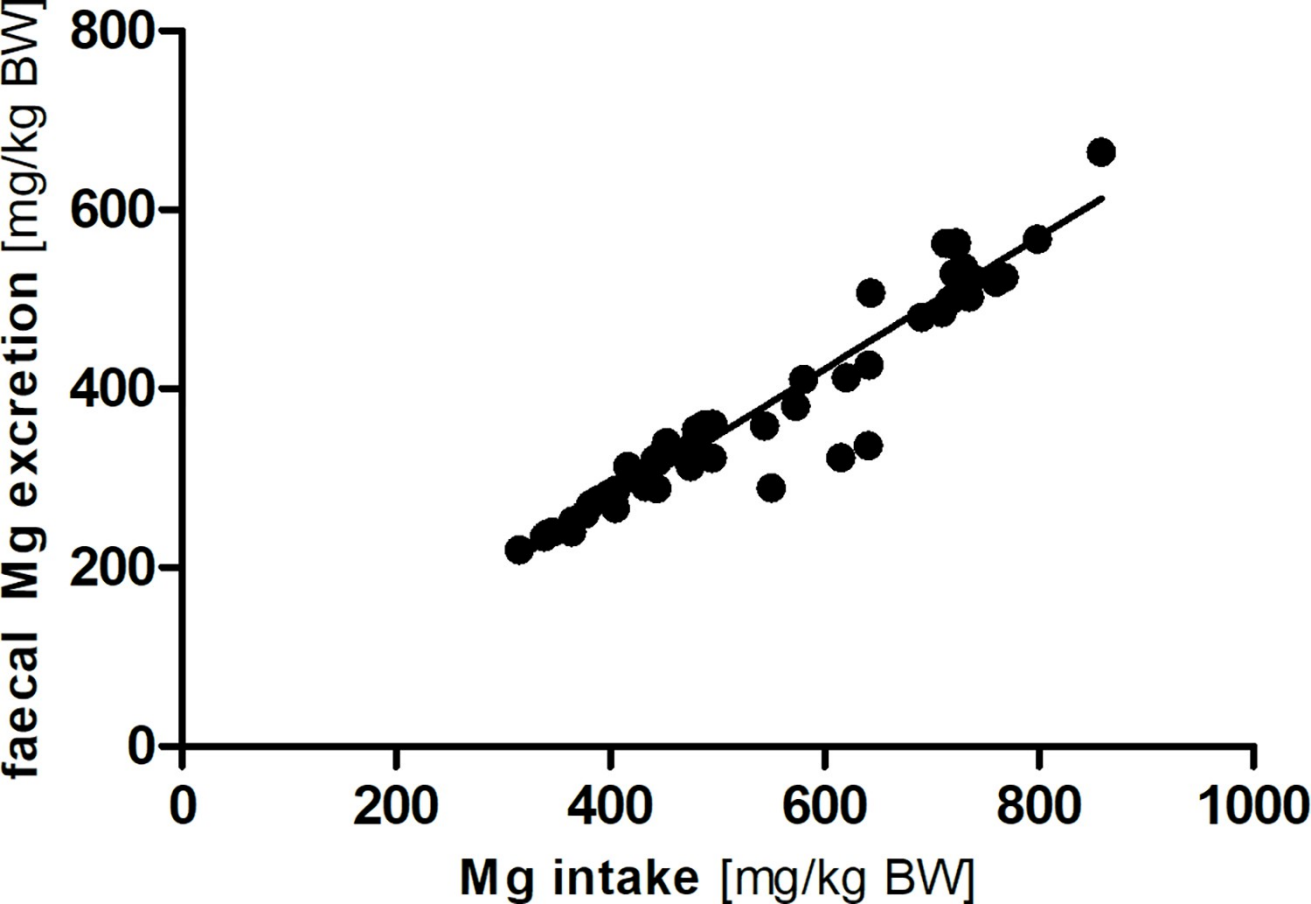
Linear correlation between magnesium (Mg) intake and faecal excretion (y = 0.74 x– 20.69; R^2^ = 0.91; Sy.x = 34).

A strong linear correlation was present between Mg intake and faecal Mg excretion in the modified Lucas-test (both mg/kg BW; R^2^ = 0.91; Sy.x = 34; Fig 6).

## Discussion

The diets fed in the present study supplied minerals in the average range of standard laboratory mouse diets, meeting or exceeding the commonly used recommendations for laboratory mice [7]. To compare the mineral intake in this study with the current recommendations by the NRC [7], we calculated the intake the theoretical mineral intake with the recommended nutrient levels according to the NRC for a mouse with the mean daily feed intake (3.92 g) and mean body weight (19.43 g) from this study. This amounted to 1,009 mg calcium, 605 mg phosphorus, 101 mg sodium, 403 mg potassium and 101 mg magnesium per kg BW. The actual mineral intake with the standard diets used in our study was considerably higher for all investigated nutrients (Table 2). However, the recommendations are based on single estimations and “acceptable performance” under conventional conditions and the NRC states that different conditions may require higher nutrient supply. A scientifically valid calculation of requirements is missing, so that the comparison between actual intake and NRC recommendation can only be an observation and no classification as excess. It should be noted that the mineral content of the diets used in this study is well within the range of commercially available standard laboratory mouse diets that are fed to mice in many facilities without obvious signs of inadequate nutrition.

In addition to the variation in dry matter digestibility of the diets [11], the amount of mineral intake differed considerably between all diets (Table 2). Thus, the overall dataset gives a range of regular mineral intake that is valuable for the performed regression analyses as a first step towards a factorial calculation of nutrient requirements. Further trials with lower and higher supply of each nutrient to extrapolate to a valid estimation of the endogenous losses that are necessary to calculate the maintenance requirement.

For all statistical procedures, nutrient intake and faecal excretion were related to body weight. In the available literature, both the absolute body weight [1, 13, 14] and the metabolic body weight (kg^0.75^) [2, 3] have been used as reference dimension in literature. Since the low average weight of laboratory mice of 20–30 g, we decided to use the body weight in this case. Regarding mineral digestibility, differences between males and non-reproducing females are not known, so that the use of female mice in this study does not pose a significant limitation.

Apparent calcium digestibility in the C57Bl/6J mice in this study was lower than reported in rats by Urbano et al. (2002) [19] and Frommelt et al. (2014) [20] in the respective control diet groups, but similar to the values given by Frischte in rats (2012) [21] and Hommel (2012) in degus (*Octodon degus*) fed a low calcium diet [22]. This may be attributed to species differences or dietary factors that lead to the variation in apparent calcium digestibility. Calcium intake and faecal calcium excretion showed a clear linear correlation. According to the slope of the regression equation of 0.67, the mean true digestibility of calcium is 33%. The linear relationship indicates that calcium excretion, and conversely absorption, is determined majorly by calcium intake (R^2^ = 0.77). In the range of calcium supply as given in the present study, no regulation of calcium absorption seems to be present. For cats and dogs, the same observation has been published [1, 2] based on large literature datasets covering a wide variety of diets and intake levels. In mice, there is evidence for vitamin D-regulated changes in intestinal calcium absorption when a low-calcium diet is consumed for a longer duration [23]. Dietary and genetic influences on calcium absorption have also been shown [24]. If mice are truly able to up-regulate intestinal absorption of calcium to a significant degree, low calcium intakes should alter the regression plot into a nonlinear model. In this case, the slope of calcium intake to faecal calcium excretion would be expected to be lower than the slope at adequate intake range.

With a range of 29 to 37%, apparent phosphorus excretion in the mice was lower than reported in rats by Frommelt et al. (2014) [20], in the same range as the rats of Fritsche (2012) [21], but much higher than in rabbits [25] and degus [22]. For phosphorus, the linear correlation between intake and faecal excretion was even stronger (R^2^ = 0.93) than for calcium in the C57Bl/6J mice, with an estimated mean true digestibility of 36%. Therefore, the major determinant for faecal phosphorus excretion seems to be phosphorus intake, without discernible regulation of intestinal absorption. This is true at least for the range of phosphorus intake observed–the present dataset does not allow deductions for a lower / below-requirement phosphorus intake. The different levels of inositol phosphates in the tested diets (Table 1) apparently had no influence on phosphorus accretion. There may be effects of higher levels of inositol phosphates limiting phosphorus availability in mice as known from other non-ruminant species like pigs and poultry [26–28]. Specific experiments with graded levels of inositol phosphates at marginal total phosphorus supply would be necessary to identify a potential relationship. With the data from the present study it is not possible to determine the renal phosphorus excretion. In some species, it is known that renal phosphorus excretion increases with increasing phosphorus intake and absorption, respectively, and contributes to the whole-body phosphorus homeostasis. This may be worth further investigation in mice.

Faecal excretion of calcium and phosphorus were closely linked in a linear regression (Fig 3; y = 0.79 x– 171.07; R^2^ = 0.75). A meta-analysis on different species groups [2] showed such a linear relationship for carnivores (i.e. cats and dogs, slope 0.51). The higher slope in this study on mice indicates a relatively higher faecal phosphorus excretion in relation to faecal calcium excretion as found in carnivores. This may be an effect of different mineral sources in the species groups, with phosphorus in the lab mouse diets possibly having a higher amount of phytate-bound phosphorus from cereal than the general carnivore diet. It is known from e.g. pigs and poultry that phytate-bound phosphorus is less available [26–28] than other phosphorus sources. From the present data, the reason for the difference cannot be determined exactly. The omnivores in the cited study (pigs and rats) had a similar slope of the regression line (0.78), but with less linearity (R^2^ = 0.53). The linear relationship between faecal calcium and phosphorus suggests that the absorption of these minerals is linked closely. As suggested previously in carnivores, this may be in part due to the formation of calcium-phosphorus complexes in the gastrointestinal tract, limiting the solubility and availability of both elements.

Apparent sodium digestibility ranged from 64 to 86%, which is lower than values reported in rats [19, 21] and pigs [29], but in a similar range as in horses [30, 31] and rabbits [25]. The linear relationship between sodium intake and apparently digested sodium (Fig 4) indicates that a fixed percentage of sodium intake is absorbed in the studied range. The mean true digestibility of sodium estimated according to the regression equation is 92%.

The apparent digestibility of potassium was significantly higher in diet PEL_81_ than the other tested diets (*p* < 0.05). Compared to literature data in rats [19, 21], the values observed in the mice of this study were lower. However, they fell into the range reported for rabbits on several test diets [25] and degus [22]. There was an extremely strong linear correlation between potassium intake and apparently digested amount of potassium (R^2^ = 0.98; Fig 5) with an estimated true digestibility of 76%. The high linearity of this relationship clearly shows that in the studied range of potassium intake, there is no regulation of intestinal potassium absorption.

A meta-analysis on micromineral digestibility in small and large hindgut fermenters [25] found that in those herbivores, sodium and potassium are absorbed to nearly 100% from the gastrointestinal tract with hardly any difference between the species. The sodium and potassium data from this study suggests that the grani-omnivorous mice (also hindgut-fermenting rodents) are similar in this aspect of mineral absorption.

There were unsystematic differences between the apparent digestibility of magnesium between the diet groups in this study (Table 2). With a range of 27–32%, the C57Bl/6J mice had similar digestibility values as observed in rats [21]. Magnesium intake and faecal magnesium excretion showed a linear relationship (R^2^ = 0.91; Fig 6), which shows that magnesium intake is a strong determinant of magnesium accretion independent of other dietary factors in the four diets used. A mean true digestibility of 26% was calculated from the regression equation.

A study on five-week-old C3H/HeMsNrsflCR mice [32] reported higher digestibility of calcium, phosphorus, and magnesium than found in the present study in >10-week-old C57BL/6J mice. For once, the studies used different mouse strains that may contribute to differences. The main factor that is likely to explain the difference is age, with younger and still growing individuals having a higher mineral digestibility than more mature ones. For future investigations, the use of different age groups (post-weaning, young adult, old adult) will be an interesting and important point.

The present study provides valuable information on quantitative aspects of mineral homeostasis in C57BL/6J mice fed a sufficient nutrient supply. Due to the lack of data in laboratory animals, basic digestibility values need to be established. It is necessary to explore the situation of below-requirement, marginally low and above-average mineral intakes in further experiments in order to estimate the endogenous losses. For each nutient, studies with an intake at different levels ranging from very low to very high need to be performed to obtain a dataset for the factorial calculation of requirements.

## Conclusions

Data on mineral digestibility in adult C57Bl/6J mice under maintenance conditions were evaluated via Lucas-test and its modification using four diets differing in form (pelleted vs. extruded), starch gelatinization as well as starch and fibre content. The digestibility of calcium, phosphorus, sodium, potassium, and magnesium remained rather uniform. This suggests that in the given range of sufficient supply, the intake of a mineral will be the main determinant of its apparently digested amount, with a fixed percentage of apparent digestibility.
